# Regeneration of *Centella asiatica *plants from non-embryogenic cell lines and evaluation of antibacterial and antifungal properties of regenerated calli and plants

**DOI:** 10.1186/1754-1611-5-13

**Published:** 2011-10-12

**Authors:** Yamin Bibi, Muhammad Zia, Sobia Nisa, Darima Habib, Abdul Waheed, Fayyaz M Chaudhary

**Affiliations:** 1 Department of Microbiology, Quaid-i-Azam University, Islamabad, Pakistan; 2 Department of Biotechnology, Quaid-i-Azam University, Islamabad, Pakistan; 3 School of Pharmacy and Chemistry, Kingston University, UK

**Keywords:** Antimicrobial, Centella asiatica, organogenesis, plant growth regulators

## Abstract

**Background:**

The threatened plant *Centella asiatica L*. is traditionallyused for a number of remedies. In vitro plant propagation and enhanced metabolite production of active metabolites through biotechnological approaches has gained attention in recent years.

**Results:**

Present study reveals that 6-benzyladenine (BA) either alone or in combination with 1-naphthalene acetic acid (NAA) supplemented in Murashige and Skoog (MS) medium at different concentrations produced good quality callus from leaf explants of *C. asiatica*. The calli produced on different plant growth regulators at different concentrations were mostly embryogenic and green. Highest shoot regeneration efficiency; 10 shoots per callus explant, from non-embryogenic callus was observed on 4.42 μM BA with 5.37 μM NAA. Best rooting response was observed at 5.37 and 10.74 μM NAA with 20 average number of roots per explant. Calli and regenerated plants extracts inhibited bacterial growth with mean zone of inhibition 9-13 mm diameter when tested against six bacterial strains using agar well diffusion method. Agar tube dilution method for antifungal assay showed 3.2-76% growth inhibition of *Mucor *species, *Aspergillus fumigatus *and *Fusarium moliniformes*.

**Conclusions:**

The present investigation reveals that non-embryogenic callus can be turned into embryos and plantlets if cultured on appropriate medium. Furthermore, callus from leaf explant of *C. asiatica *can be a good source for production of antimicrobial compounds through bioreactor.

## Background

In recent years considerable attention has been paid to utilize eco-friendly and bio-friendly plant based products, normally secondary metabolites, for the prevention and cure of different human diseases including microbial infections [1]. Moreover, the continuous and non-organized exploitation has resulted in many plants becoming rare or extinct. So there is a dire need to set a protocol for in vitro production of plants. In view of commercial importance given to secondary metabolites, efficient production of bioactive compounds by tissue culture technology has gained popularity [2]. Secondary metabolites often have a complex stereo structure and many of these cannot be synthesized economically on a commercial basis. To overcome this limitation, biotechnologists suggest "the use of cell and tissue culture technology rather than the whole plant for the extraction of certain secondary metabolites [3]. Antimicrobial potential of cell culture has been studied in a number of plants i.e. *Sesuvium portulacastrum L, Ephedra strobiliacea, Ephedra procera, Ephedra pachyclada *[4,5], and reports are also available for increased metabolite production in callus culture of *Calophyllum brasiliense *(calanolides)*, Psoralea drupacea *Bge (bakuchiol) and *Artemisia absenthium *(artemisinin) [6-8].

*Centella asiatica L*. commonly known as Indian pennywort belongs to family Apiaceae. Traditionally it is used as nervine tonic and for the treatment of asthma, hypertension, bronchitis, dropsy, skin diseases, and urethritis [9,10]. *C. asiatica *has antibacterial, antifeedant, antituberculosis, antileprotic, and antioxidant properties [11-13]. Glycosides like indocentelloside, brahmoside, brahminoside, asiaticoside and theankuniside have been isolated from this plant. Asiaticoside is used in treatment of leprosy and tuberculosis [14].

Multiplication of *C. asiatica *through shoot tip culture [15] and meristem tip culture [14] has been reported. Stimulation of asiaticoside accumulation in whole plant cultures of *C. asiatica *by elicitors including yeast extract, CdCl_2_, CuCl_2 _and methyl jasmonate has been studied, and methyl jasmonate achieved maximum asiaticoside production [16]. Although callus culture and regeneration of *C. asiatica *has already been established, the present study assessed embryogenic response of non-embryogenic callus and to evaluate the antibacterial and antifungal potential of highly proliferated calli and regenerated plants.

## Results and discussion

**Figure 1:**
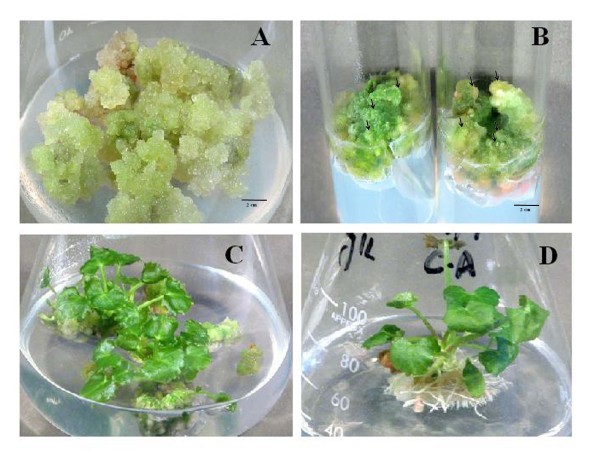
**Callogenesis and organogenesis of *Centella asiatica *L. from leaf explant on MS media containing; (A) callus formation on 5.37 μM NAA+2.21 μM BA; (B) callus formation on 5.37 μM NAA+4.42 μM BA; (C) shooting at 4.42 μM BA+5.37 μM NAA; (D) rooting at 10.74 μM NAA**.

Leaf explants of *Centella asiatica *developed callus at cut surfaces and subsequently covered the entire surface of explant within 15-20 days. Callogenic response was observed on all media except the media without growth regulator. Media containing low concentration of naphthalene acetic acid (NAA; 2.68 μM) or low/high concentration of 6-benzyladenine (BA; 2.21 and 17.68 μM) also was not supportive for callus induction. Green, compact and embryogenic calli were observed with 66-100% response at BA and NAA different concentrations. Most of the media showing rapid growth of callus contained both an auxin (i.e., NAA) and a cytokinin (BA). The callus produced was compact and embryogenic with variation in colour (Table 1). Combination of NAA and BA at varying levels also resulted in difference in callogenic response (Figure 1A &1B). Only at two combinations (10.74 μM NAA + 4.42 μM BA and 21.48 μM NAA + 4.42 μM BA) was the callus non-embryogenic, friable and light green to brownish green (Figure 1A). Our results contradict Patra et al. [17] who reported that addition of NAA in MS medium containing BA or kinetin decreased callus formation response. While the best callogenic response from leaf explants was observed on MS medium supplemented with BA or Kinetin along with 2,4 dichlorophenoxy acetic acid, good callus formation was not observed when media was supplemented with BA and NAA [18]. Calli characteristics and induction response vary depending on explant source, explant size, media components and presence of plant growth regulator in media along with other culture conditions [8,19]. We observed that the embryogenic callus became hard after few days and embryo-like structures could be observed. In the present study, the embryogenic calli produced shoots after remaining on the same medium for an additional 2-4 weeks. However, due to our focus on production of shoots from non-embryogenic callus data were not recorded. The callus which failed to produce embryos (non-embryogenic) proliferated at much faster rate as compared with embryogenic calli in the same time period.

**Table 1:** Effect of growth regulators on callus induction from *Centella asiatica *leaf explants

Plant Hormone	Concentration (μM)	Proliferation rate	Response (%)	Callus characteristics
Blank	NIL	-	-	-
NAA	2.68	-	-	-
NAA	5.37	+	75 ± 4.2^c^	Green, compact, embryogenic
NAA	10.74	++	66 ± 6.7^d^	Green, compact, embryogenic
NAA	21.48	+++	100 ± 4^a^	Green, compact, embryogenic
BA	2.21	-	-	-
BA	4.42	+++	66 ± 7.1^d^	Green, compact, embryogenic
BA	8.84	+++	100 ± 3.1^a^	Green, compact, embryogenic
BA	17.68	-	-	-
NAA+BA	2.68+2.21	+++	100 ± 3.5^a^	Lush green, compact, embryogenic
NAA+BA	5.37+2.21	++	100 ± 4.6^a^	Granular, light green, nonembryogenic
NAA+BA	10.74+2.21	+	40 ± 8.2^e^	Brownish, very small
NAA+BA	21.48+2.21	++	92 ± 4.9^b^	Compact, green, embryogenic
NAA+BA	2.68+4.42	++++	89 ± 3.7^b^	Compact, green, embryogenic
NAA+BA	5.37+4.42	++++	100 ± 2.1^a^	Compact, green, embryogenic
NAA+BA	10.74+4.42	++	83 ± 4^c^	Friable, brownish green, non embryogenic
NAA+BA	21.48+4.42	++	88 ± 2.8^b^	Friable, brownish green, non embryogenic
NAA+BA	2.68+8.84	+++	88 ± 3.3^b^	Compact, whitish green
NAA+BA	5.37+8.84	++++	100 ± 2.1^a^	Lush green, compact, embryogenic
NAA+BA	10.74+8.84	++++	88 ± 5^b^	Lush green, compact, embryogenic
NAA+BA	21.48+8.84	+	88 ± 8^b^	Compact, brownish green, embryogenic
NAA+BA	2.68+17.68	++	83 ± 7.1^c^	Lush green, compact, embryogenic
NAA+BA	5.37+17.68	++++	100 ± 3.4^a^	Dark green, compact, embryogenic
NAA+BA	10.74+17.68	++++	79 ± 5.7^c^	Whitish green, compact, embryogenic
NAA+BA	21.48+17.68	+++	100 ± 1.9^a^	Greenish white, compact, embryogenic

Percent response means followed by the letter are significantly different at the 0.05 level of confidence

Non-embryogenic, light green and granular callus produced on 5.37 μM NAA with 2.21 μM BA was shifted to shooting media consisting of different hormonal combinations but response appeared only in two combinations (BA+NAA; 2.21+5.37 μM and 4.42+5.37 μM) after about 8 weeks (Table 2). Generally low auxin and high cytokinin concentrations in the media result in induction of shoot morphogenesis [20]. Our contrary observations can be explained by classical findings of Skoog and Miller [21] that organogenesis in tissue culture is governed by the balance of auxin and cytokinin in the media. Conversion of non embryogenic callus into embryos and organs is not well understood. However, the presence of plant hormone and its concentration sequent the cell for differentiation. Cell mass became hard and compact after few days, and dome-like structures could be observed on the surface presenting formation of embryos. Fruthermore, long time culture of callus on same media produces embryos although the response remains low [22]. The embryogenic callus produced greater number of shoots in less time period (data not shown). Conversion of dome-like structures (in embryogenic callus) into shoots over hormone dependent maturation has been studied extensively in many plants including *C. asiatica *[18]. Maximum 5.3 shoots per node with 91% efficiency was observed through direct shoot regeneration from nodal segments of *C. asiatica *on BA- and NAA- containing medium [14]. In the present study, when the calli remained on the same medium for long time, a maximum of 10 ± 6.1 shoots were observed.

**Table 2:** Effect of growth regulators on shoot regeneration from non-embryogenic callus of *Centella asiatica*

Plant hormone	Concentration (μM)	Response (%)	Number of Shoots per callus
BA	0	0	0
BA	4.42	0	0
BA	8.84	0	0
BA	17.68	0	0
BA+NAA	2.21+5.37	50	4 ± 2.5
BA+NAA	4.42+5.37	50	10 ± 6.1
BA+NAA	8.84+5.37	0	0
BA+NAA	17.68+5.37	0	0
Kin+NAA	4.65+5.37	0	0
Kin+NAA	9.29+5.37	0	0
Kin+NAA	23.23+5.37	0	0

Regenerated shoots were green, leafy and multiple as shown in Figure 1C. The shoots were separated and transferred to rooting medium to form complete plants. Best rooting response (20 roots per plantlet) was observed in the medium containing high concentration of NAA (5.37 μM and 10.74 μM) after 20 days (Table 3). Roots were white and thin as shown in Figure 1D. Patra et al. [17] also observed the same rooting response with NAA in *C. asiatica*.

**Table 3:** Rooting response from *Centella asiatica *regenerated shoots on different hormonal concentrations

Plant hormone	Concentration (μM)	Response (%)	No of roots per shoot
NAA	2.68	0	0 ± 0
NAA	5.37	100	20 ± 0.8^a^
NAA	10.74	100	20 ± 0.5^a^
IBA	2.46	0	0 ± 0
IBA	4.92	100	7 ± 0.6^b^
IBA	9.84	50	3 ± 0.2^c^

Means number of roots per shoot followed by the letter are significantly different at the 0.05 level of confidence

Rooted plantlets were separated from medium washed and extracted in methanol. Highly proliferated calli on NAA+BA (10.74+4.42 μM and 5.37+17.68 μM, respectively) were also extracted in methanol. Dried extracts were tested for antibacterial activity. Callus extracts were active against all six tested organisms. Callus extracts showed maximum activity against *Pseudomonas picketii *with mean zone of inhibition 15 mm. The calli proliferated on high concentration of BA and low NAA (10.74+4.42 μM) was more active against *Escherichia coli *while it showed equal zone of inhibition against *Micrococcus leutus *and *Bacillus subtilis *as compared with calli regenerated/proliferated on low BA and high NAA. Shahid et al. [23] also reported that calli extract of *Saraca asoca *were more active against Gram negative and Gram positive bacteria as compared with whole plant extract while Landa et al. [24] concluded that the extracts of *Nigella *species calli tested exhibited significant anti-microbial activity, especially against *Bacillus cereus, Staphylococcus aureus *and *Staphylococcus epidermidis*.

**Figure 2:**
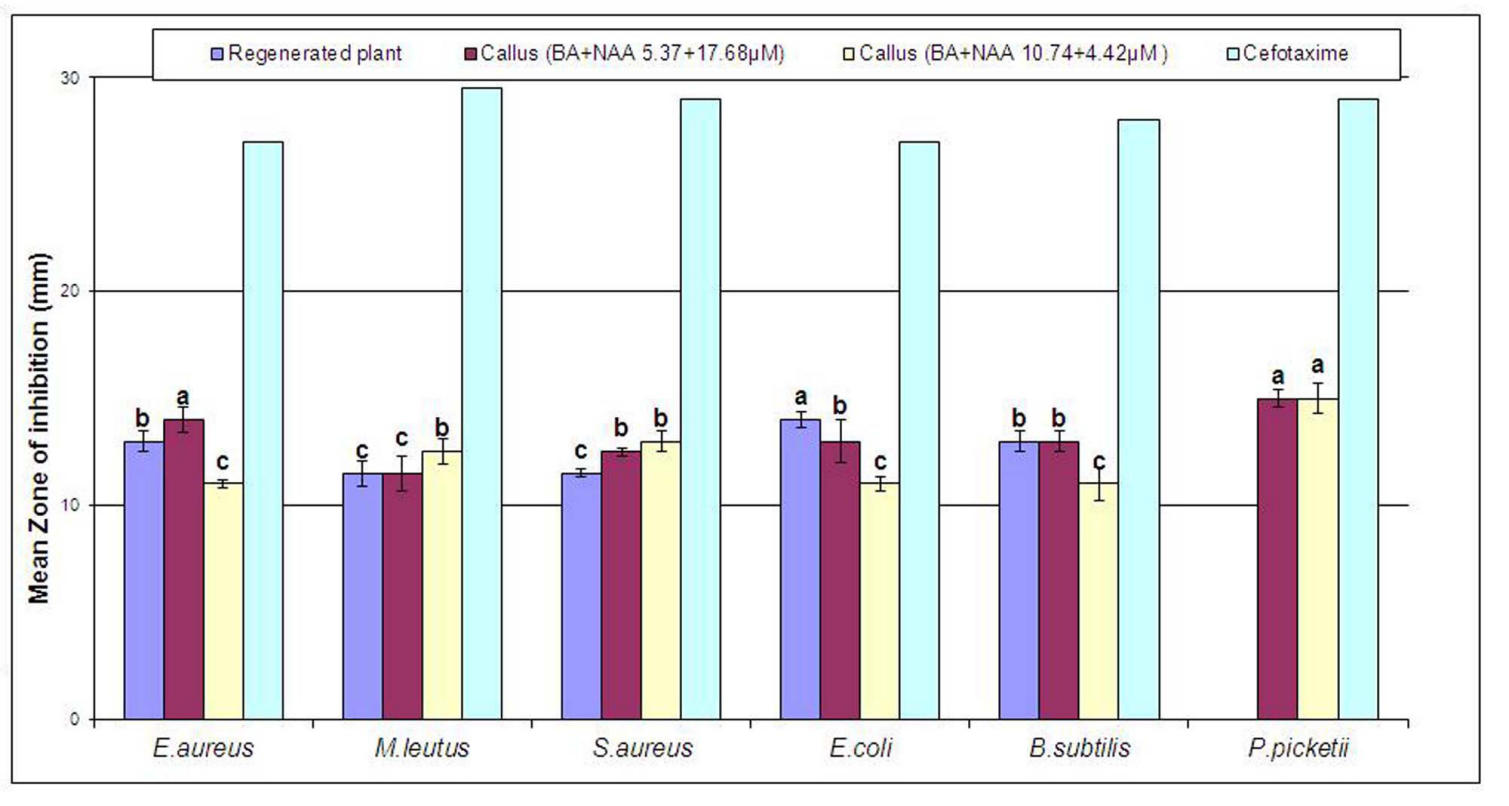
**Antibacterial activity of regenerated plants and callus of *Centella asiatica *L**. Results are average of triplicate. Letters on bars represent statistical difference in values (LSD) at the 0.05 level of confidence.

Regenerated plant extracts were active against all strains except *P. picketii*, and showed equal or lower activity compared to callus extracts against all strains except *E. coli *(Figure 2). The maximum activity shown by regenerated plants extract was against *E. coli *with mean zone of inhibition 14 mm. This result contradicts previous findings of Panthi and Chaudhary [25] related to antibacterial activity of *in vivo *plants of *C. asiatica *which showed activity against many strains but not *E. coli*. Also, Ullah et al. [26] reported inhibition zone up to 15 mm against some bacteria by different fractions of *C. asiatica *extract. These differences might be due to different culture conditions such as concentrations of growth regulators which affect active metabolite yield and hence difference in bioactivities [27].

**Figure 3:**
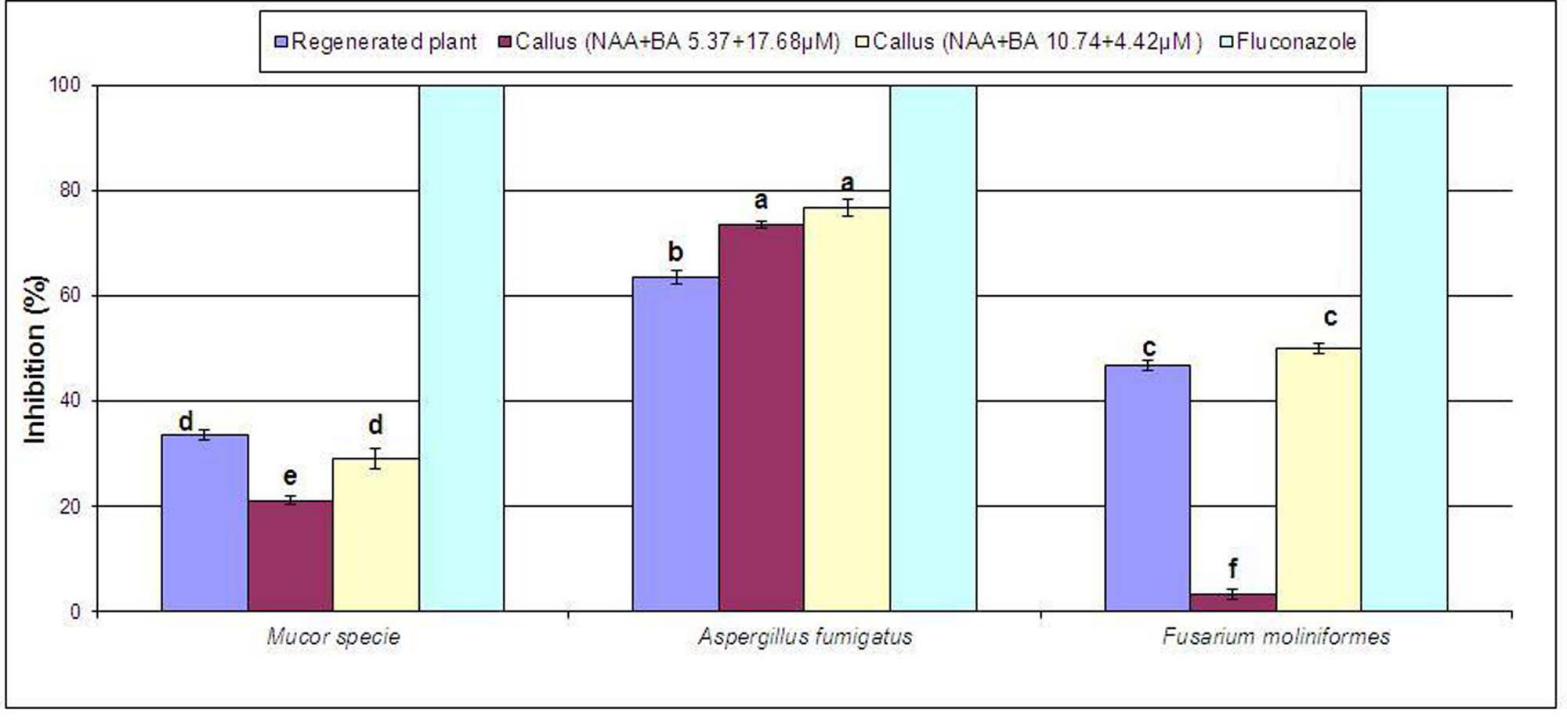
**Antifungal activity of regenerated plants and callus of *Centella asiatica *L**. Results are average of triplicate. Letters on bars represent statistical difference in values (LSD) at the 0.05 level of confidence.

Dried extracts were also tested for antifungal activity. Both the callus extracts as well as regenerated plants extract were active against all three tested organisms, and maximum activity was observed against *Aspergillus fumigatus *with inhibition of 73.4% and 76.7%. Regenerated plant extract showed less activity compared with callus extracts against all strains except *Mucor *species (Figure 3). Though, production of bioactive metabolites in cell or callus culture is usually low as compared to wild grown plants [28]. However plant growth regulators may triggers different biochemical pathways in cell, result in enhanced production of metabolites. In present study near about equal activities were observed by calli extracts as compared with reported bioactivities of *C. asiatica *plant extract [26].

## Conclusions

In conclusion, *Centella asiatica *non-embryogenic callus can be converted to embryogenic and shoots when cultured on appropriate concentrations of BA and NAA in MS medium. Both the regenerated plant and callus extracts inhibited bacterial and fungal growth. Therefore, active metabolites produced through callus might serve as an alternative source of bioactive compounds, in order to reduce the pressure on wild stocks.

## Methods

### Plant material

Plants of *Centella asiatica *were collected from the bank of Rawal Dam Islamabad Pakistan. A voucher specimen was deposited in the Herbarium of Quaid-i-Azam University Islamabad Pakistan. Plants were dipped in 5% detergent solution (Max v/v) for 5 min and then thoroughly washed under running tap water for 30 min. Under aseptic conditions, leaves were separated from stems and surface sterilized in 0.1% (w/v) mercuric chloride solution for 1-2 min. After 4-5 rinses with sterilized distilled water, leaves were placed on sterilized filter papers to remove excess moisture. Leaves were cut into small segments of 8 mm^2 ^to be used as explants.

### Callogenesis

Basal Murashige and Skoog (MS) media [29] supplemented with different concentrations of 6-benzyladenine (BA; 2.21-17.68 μM) and nephthalene acetic acid (NAA; 2.68-21.48 μM) either alone or in combination were used for callus induction from leaf explant of *C. asiatica*. The pH of media was adjusted at 5.7 with 0.1N HCl and NaOH before autoclaving. Sucrose (3%) was added as carbon source and Nobel agar (0.7%) as solidifying agent. A volume of 40-50 ml was dispensed in magenta jars or 100 ml conical flasks. The flasks/jars containing media were sterilized under 15 psi pressure at 121°C for 20 min. Under aseptic conditions leaf explants were inoculated on the surface of media. The flasks were incubated at 25°C and 16/8 light dark period in a growth room. The test was performed in triplicate; each replicate contained 10 explants and data were recorded after 30 days of inoculation.

Callus cultures were maintained on MS medium containing same growth regulators at 25°C with a daily photoperiod of 16 h. In order to produce enough biomass, calli were sub-cultured on monthly basis on the fresh medium. Phenotypic callus characteristics such as colour, friability and proliferation rate were assessed at every cycle during the maintenance stage.

### Organogenesis

To determine shooting response, non-embryogenic callus produced on MS medium containing 5.37 μM NAA and 2.21 μM BA was selected. The callus was granular (scattered cells), friable and no dome-like structures were observed which appeared to be non-embryogenic under microscopic examination. The callus was transferred to shooting medium (MS) supplemented with 3% sucrose and BA (0-17.68 μM), NAA (5.37 μM), and Kinetin (Kin; 4.65-23.23 μM) either alone or in combinations. The pH was maintained at 5.7 and the media was solidified by adding 0.7% Nobel agar. These cultures were maintained at 25°C with 16 h daily photoperiod. The explants were transferred on fresh medium after 2-3 weeks. The test was performed in triplicate. Each replicate contained six explants, and data were recorded after 60 days of culture.

When heights attained 3-4 cm, the shoots transferred to rooting medium composed of MS medium supplemented with different concentrations of NAA (2.68-10.74 μM) and indol 3-butyric acid (IBA; 2.46-9.84 μM)). The medium was also supplemented with 3% sucrose, 0.7% Nobel agar and the pH was adjusted at 5.7. The test was performed in triplicate. Each replicate contained six explants, and the data were recorded after 20 days of culture. The rooted plants were washed under running tap water to remove excess media and transferred to vermiculite-containing pots. The pots were covered with transparent polythene bags to retain humidity. The plants were watered with Hogland solution when required. After one week, the polythene bags were gradually removed, and plants were transferred to pots containing peat moss and clay (1:1) and shifted to green house.

### Extraction

Regenerated whole plants (34 g FW) and proliferated calli (47 g FW) on two hormonal combinations (NAA+BA; 10.74+4.42 μM and 5.37+17.68 μM) were ground in 100 ml methanol and maintained at room temperature for seven days. The extract was subsequently filtered through Whatman filter paper No.1. The residue was again dipped in methanol for additional seven days. Finally, the extracts were combined and concentrated using rotary evaporator at low pressure. Obtained plant and callus extracts (2 g and 1.3 g, respectively) were subjected for antimicrobial potential.

### Antibacterial activity

Bacterial strains used to assess antimicrobial activity were *Enterococcus aureus, Staphylococcus aureus, Micrococcus leutus, Escherichia coli, Bacillus subtilis *and *Pseudomonas picketii*. Stock cultures were maintained on nutrient agar slants at 4°C and then subcultured in nutrient broth at 37°C prior to antibacterial test. Dried extracts of callus and plants at the concentration 25 mg/ml were tested for antibacterial activity using agar well diffusion method [30]. Cefotaxime at a concentration of 2 mg/ml was used as positive control. Pure dimethyl sulfoxide (99.9% DMSO) solvent was used as negative control. Nutrient agar medium was prepared by suspending 20 g/l nutrient agar (Merck) and autoclaved. When the medium cooled down at 45°C, it was seeded with 10 ml of 24 h old bacterial culture containing approximately 10^4^-10^6 ^colony forming units per ml. Petri plates were prepared by pouring 75 ml of seeded nutrient agar per plate. Wells were made with 8 mm cork borer, and each well was sealed with 20 μl of molten nutrient agar. Experimental plates were incubated at 37°C for 24 h and zones of inhibition (mm) were measured and compared with standard antibiotic zone of inhibition.

### Antifungal activity

Fungal strains used were *Mucor *species, *Aspergillus fumigatus *and *Fusarium moliniformes*. Stock cultures were maintained on nutrient agar slants at 4°C and then subcultured in Sabouraud Dextrose agar slants at 28°C seven days prior to antifungal assay.

The antifungal activity of calli and regenerated plants extracts was determined using the agar tube dilution method [31]. Sabouraud dextrose agar (Merck) was dissolved and dispensed as 4 ml per screw capped tube and autoclaved at 121°C, 15 psi pressure for 20 min. The tubes were allowed to cool at 50°C, and 100 μl of plant extract was poured from the stock solution (20 mg/ml in DMSO) to attain a final concentration of 50 μg/ml. Fluconazole (12 mg/ml) was used as positive control, DMSO was a negative control. The tubes were shaken well and allowed to solidify at slanting position at room temperature. The tubes were then inoculated with 4 mm piece of inoculum and incubated at 28°C for 7 days. Growth was determined by measuring linear growth (mm), and growth inhibition was calculated with reference to negative control using following formula

$$\text{Percentage inhibition of fungal growth}=100-\left[\text{Linear growth in test tube }\left(\text{mm}\right)∕\text{Linear growth in control }\left(\text{mm}\right)\right]\times 100$$

### Statistical analysis

To analyze callogenic and organogenic response, the tests were performed in triplicate for each hormone concentration/combination. Each replicate contained 10 explants for callogenesis and six explants for organogenesis (shooting and rooting separately). Callogenic data were recorded after 30 days of culture and analysed using Least Significant Difference (LSD) at probability level p < 0.05. Shooting response was analyzed for average number of shoots per explant after 60 days of culture. Rooting data recorded after 20 days of culture were analysed for average number of roots per plantlet by t-test at probability level p < 0.05 for least significant difference.

The antibacterial and antifungal tests were performed in triplicate for each microorganism. The means of average zone of inhibition in antibacterial assay and growth inhibition for antifungal assay were further analysed using LSD at probability level p < 0.05.

## List of abbreviations

BA: 6-benzyladenine; DMSO: dimethyl sulfoxide; IBA: indole 3-butyric acid; Kin: kinetin; LSD: Least significant difference; MS: Murashige and Skoog; NAA: nephthalene acetic acid

## Competing interests

The authors declare that they have no competing interests.

## Authors' contributions

YB, SN, DH and AW carried out the experimental part such as callogenesis, organogenesis, preparation of inoculums, antibacterial and antifungal assay. MZ evaluated the results, and wrote the manuscript. FMC supervised the work and corrected the manuscript. Authors read and approved the final manuscript.
